# Granulocyte Macrophage Colony-Stimulating Factor-Activated Eosinophils Promote Interleukin-23 Driven Chronic Colitis

**DOI:** 10.1016/j.immuni.2015.07.008

**Published:** 2015-07-21

**Authors:** Thibault Griseri, Isabelle C. Arnold, Claire Pearson, Thomas Krausgruber, Chris Schiering, Fanny Franchini, Julie Schulthess, Brent S. McKenzie, Paul R. Crocker, Fiona Powrie

**Affiliations:** 1 Kennedy Institute of Rheumatology, Nuffield Department of Orthopaedics, Rheumatology and Musculoskeletal Sciences, University of Oxford, Roosevelt Drive, Oxford OX3 7FY, UK; 2 Translational Gastroenterology Unit, Experimental Medicine Division Nuffield Department of Clinical Medicine, University of Oxford, John Radcliffe Hospital, Oxford OX3 9DU, UK; 3 CSL Ltd. Research Department, Bio21 Molecular Science and Biotechnology Institute, University of Melbourne, 30 Flemington Road, Parkville, Victoria 3010, Australia; 4 Division of Cell Signalling and Immunology, College of Life Sciences, University of Dundee, Dundee DD1 5EH, UK

## Abstract

The role of intestinal eosinophils in immune homeostasis is enigmatic and the molecular signals that drive them from protective to tissue damaging are unknown. Most commonly associated with Th2 cell-mediated diseases, we describe a role for eosinophils as crucial effectors of the interleukin-23 (IL-23)-granulocyte macrophage colony-stimulating factor (GM-CSF) axis in colitis. Chronic intestinal inflammation was characterized by increased bone marrow eosinopoiesis and accumulation of activated intestinal eosinophils. IL-5 blockade or eosinophil depletion ameliorated colitis, implicating eosinophils in disease pathogenesis. GM-CSF was a potent activator of eosinophil effector functions and intestinal accumulation, and GM-CSF blockade inhibited chronic colitis. By contrast neutrophil accumulation was GM-CSF independent and dispensable for colitis. In addition to TNF secretion, release of eosinophil peroxidase promoted colitis identifying direct tissue-toxic mechanisms. Thus, eosinophils are key perpetrators of chronic inflammation and tissue damage in IL-23-mediated immune diseases and it suggests the GM-CSF-eosinophil axis as an attractive therapeutic target.

## Highlights

• GM-CSF synergizes with IL-5 to exacerbate eosinopoiesis during chronic colitis
• GM-CSF-activated eosinophils promote IL-23 driven colitis
• Depletion of eosinophils, but not of neutrophils, dampens colitis
• GM-CSF increases eosinophil production of inflammatory cytokines TNF and IL-13

## Graphical Abstract

**Figure:**
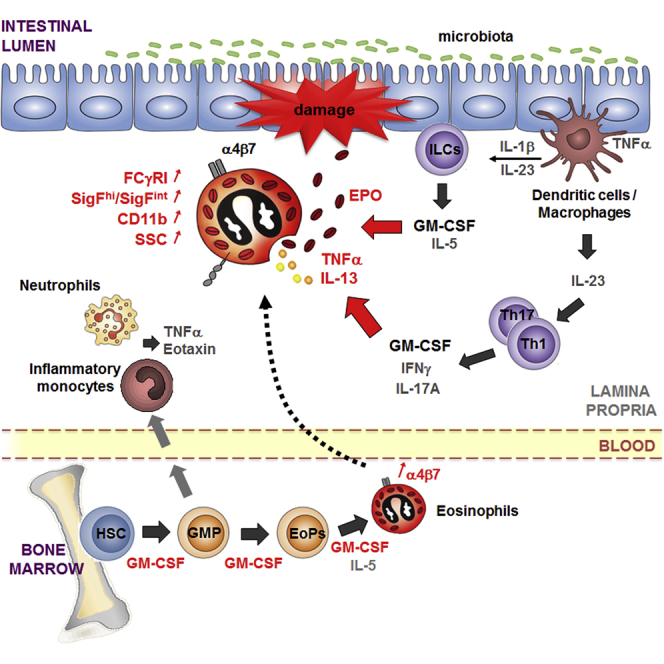

## Teaser

Although eosinophils are most commonly associated with Th2-cell-mediated diseases, Powrie and colleagues describe a crucial role for eosinophils in IL-23-mediated chronic colitis. GM-CSF was a potent activator of eosinopoiesis and concomitant accumulation of activated and tissue-toxic eosinophils in the inflamed intestine, as well as their secretion of inflammatory cytokines.

## Introduction

Chronic intestinal inflammation is characterized by dysregulated T helper 1 (Th1) and Th17 cell and innate lymphoid cell responses with excessive production of inflammatory cytokines (Maloy and Powrie, 2011), leading to increased production of granulocyte-monocyte progenitors (GMPs) and accumulation of inflammatory myeloid cells in the target tissue (Griseri et al., 2012). Previously we described an interleukin-23 (IL-23)-granulocyte macrophage colony-stimulating factor (GM-CSF) axis as a key driver of dysregulated hematopoiesis in colitis (Griseri et al., 2012); however, the relative contribution of distinct innate effector cells downstream of this pathway remains unknown. Neutrophils are considered a major culprit in IL-23-Th17-cell-type-mediated tissue damage (Chin and Parkos, 2006; Nathan, 2006), while the pathogenic role of eosinophils has primarily been established for Th2 cell-mediated conditions such allergic skin and lung disease (Rosenberg et al., 2013).

Eosinophils, which arise from GMPs through an eosinophil progenitor (EoP) intermediate (Iwasaki et al., 2005), are rare in the blood but more abundant in tissues such as the gastrointestinal tract, although their contribution to intestinal homeostasis remains enigmatic (Kita, 2011; Mishra et al., 1999). Beyond their role in Th2 cell immunity, eosinophil secrete various inflammatory mediators (e.g., TNF, IL-13, CXCL1) and have been implicated in activation of dendritic cells (DCs) and neutrophils (Rosenberg et al., 2013). They can also release anti-microbial compounds toxic for viruses and bacteria and promote the survival of immunoglobulin A (IgA)-secreting plasma cells in the intestine, suggesting a possible anti-microbial function (Chu et al., 2014; Rosenberg et al., 2013).

A dysregulated eosinophil response can cause immune pathology, and this is most evident in atopic diseases such as asthma and eczema, Th2 cell-mediated eosinophilic esophagitis, and hypereosinophilic syndrome (Fulkerson and Rothenberg, 2013). However, the molecular signals that drive eosinophils from protective to tissue damaging cells are ill-defined and require further characterization. Similar to neutrophils, eosinophils produce a range of cytotoxic mediators; matrix metalloproteinases and reactive oxygen species, as well as specific proteins such as eosinophil peroxidase (EPO) and eosinophil cationic protein (ECP) (Fulkerson and Rothenberg, 2013). These molecules are toxic for invading microorganisms but can also lead to collateral damage to host tissues including the intestinal epithelium (Fulkerson and Rothenberg, 2013; Plager et al., 2009). Indeed, intestinal eosinophil accumulation has been implicated in the pathogenesis of a chemically induced model of acute colonic injury (Forbes et al., 2004) and increased eosinophil numbers and activation has been reported in inflammatory bowel disease (IBD) (Ahrens et al., 2008; Saitoh et al., 1999). However, despite their abundance in the intestine, the regulation of eosinophils by colitogenic cytokines and their functional role in chronic intestinal inflammation is not known.

Our previous work identified IL-23-driven GM-CSF as a key mediator of chronic inflammation in T cell transfer colitis. GM-CSF promoted intestinal inflammation at several levels, including skewing of hematopoiesis toward granulo-monocytopoiesis and accumulation of highly proliferative GMPs in the intestine (Griseri et al., 2012). Using experimental models of chronic colitis, we now show that GM-CSF promoted IL-23-driven intestinal inflammation through local accumulation of activated eosinophils and potentiation of their effector functions. In addition, it also promoted bone marrow (BM) eosinopoiesis in synergy with IL-5. Because IL-23 is a well-known driver of the Th17 cell response, these results provide evidence of a link between the Th17-cell-type response and eosinophils in intestinal inflammation and suggest that targeting the GM-CSF-eosinophil axis might have therapeutic utility in some forms of IBD.

## Results

### Chronic Colitis Is Associated with High Numbers of Activated Eosinophils

**Figure 1:**
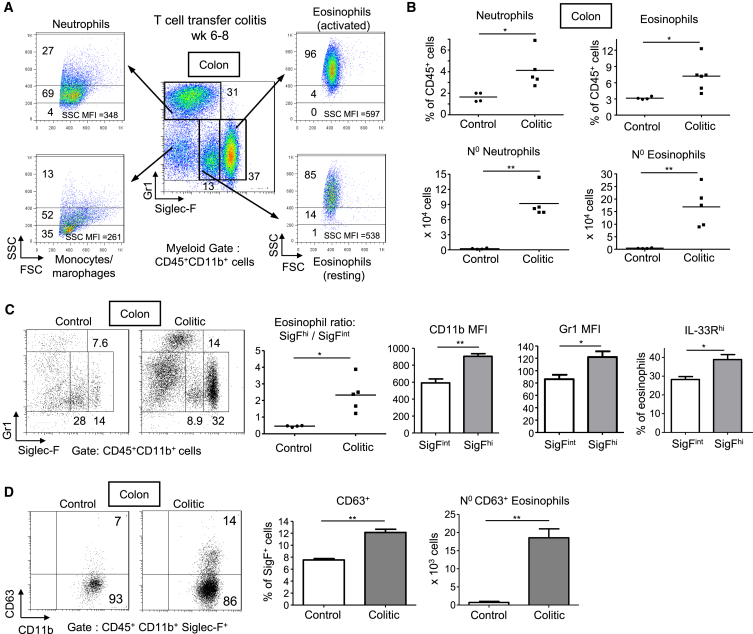
Chronic Colitis Is Associated with an Increase in Activated Eosinophils Colitis was induced by transfer of naive CD4^+^CD45RB^hi^ T cells into C57BL/6.*Rag1*^*−/−*^ mice. Mice were analyzed 6 to 8 weeks after transfer (colitic) and compared with untransferred *Rag1*^−/−^ mice (control). (A) Representative staining of resting eosinophils (Gr1^int^Siglec-F^int^SSC^hi^), activated eosinophils (Gr1^int^Siglec-F^hi^SSC^hi^), neutrophils (Gr1^hi^Siglec-F^−^SSC^int^), and monocytes or macrophages (Gr1^int^Siglec^−^F^−^SSC^lo/int^) from colonic lamina propria leukocytes (cLPL). Frequencies and MFI are indicated. (B) Frequencies and absolute numbers of neutrophils and Siglec-F^hi^ eosinophils (termed “eosinophils” thereafter). (C) Representative Gr1 and Siglec-F staining from cLPL (left). Ratio of activated/resting (SigF^hi^/SigF^int^) eosinophils in cLPL (middle). CD11b and Gr1 MFI of colonic eosinophils in colitis and frequencies of IL-33R^hi^ eosinophils (right). (D) Representative CD63 staining, frequencies, and absolute numbers of CD63^+^Siglec-F^hi^ eosinophils (±SEM, n = 5 per group). Data points represent individual mice and bars represent means (B and C). Data are representative of two to three independent experiments. See also Figure S1.

To investigate the relative contribution of granulocyte subsets to chronic intestinal inflammation, we used a well-characterized T cell transfer model of IL-23 driven colitis. In this model, chronic colitis develops ∼6 weeks after transfer of T cells into *Rag1*^−/−^ mice and is accompanied by increased granulopoiesis (Griseri et al., 2012). Standard markers were used to discriminate eosinophils (Siglec-F^+^Gr1^int^) from Siglec-F^−^Gr1^hi^ neutrophils (Dyer et al., 2011; Zhang et al., 2004). We also used the level of expression of Siglec-F as a measure of eosinophil activation. Thus, Siglec-F^int^ cells are immature or resting eosinophils that reside in lymphoid organs and in uninflamed tissues, whereas Siglec-F^hi^ cells are mature or activated eosinophils mostly found in extralymphoid tissues and increased during inflammation (Rose et al., 2010; Voehringer et al., 2007). In chronic colitis, we found both populations among colonic lamina propria leukocytes (cLPL), with high granularity (SSC^hi^) and expression of the eotaxin receptor CCR3 confirming them as eosinophils (Figures 1A and S1A). Percentages of Siglec-F^hi^ eosinophils were as high as those of neutrophils in the inflamed intestine, both of which were ∼2-fold increased in colitic compared to control *Rag1*^*−/−*^ mice. This increase was equivalent to a ∼40-fold increase in absolute numbers (Figures 1A and 1B). The abundance of intestinal eosinophils was confirmed in situ, with a high density of Siglec-F^+^ cells observed in inflamed colons (Figure S1E). IL-23-deficient *Rag1*^*−/−*^ mice, which only develop mild colitis after T cell transfer (Hue et al., 2006), had a reduced absolute number and percentage of eosinophils among CD45^+^ leukocytes compared to colitic IL-23 competent mice, suggesting a link between intestinal eosinophil accumulation and IL-23-driven inflammation (Figure S1B).

Eosinophils in the inflamed intestine showed increased activation based on a number of parameters. First the majority express high amounts of Siglec-F (Figure 1C), a phenotype associated with activation in inflammatory lung disease (Ohnmacht et al., 2007; Rose et al., 2010). Consistent with their increased activation state, Siglec-F^hi^ eosinophils in the inflamed intestine also expressed higher amounts of other eosinophil activation markers such as CD11b, IL-33R, and Gr1 (Figure 1C) (Kita, 2011; Masterson et al., 2014; Stolarski et al., 2010). These activated eosinophils in colitis also expressed the degranulation marker CD63 (Verjan Garcia et al., 2011). Such cells were 1.7-fold higher in the colon of colitic mice compared to controls with a 60-fold increase in total numbers (Figure 1D). Finally, while resting Siglec-F^int^ eosinophils in the BM and spleen secreted negligible amounts of TNF, 14% of eosinophils in the inflamed colon were positive for TNF (Figure S1D), a crucial colitogenic cytokine (Maloy and Powrie, 2011).

Interestingly, protection from colitis by co-transfer of Foxp3^+^ regulatory T (Treg) cells with colitogenic CD4^+^ T cells reduced not only cLPL neutrophil numbers but also eosinophil accumulation and activation, indicating similar inhibitory effects of Treg cells on neutrophils and eosinophils in this model (Figure S1C).

Taken together, these data show that eosinophils are a major constituent of the IL-23-driven intestinal inflammatory network (∼4 to 12% of total cLPL in colitis, Figure 1B). Although a predominant population of eosinophils in the normal intestinal mucosa had a resting Siglec-F^int^ phenotype, a shift toward an activated Siglec-F^hi^ population with signs of degranulation occurred during chronic intestinal inflammation.

### Increased Eosinopoiesis Is a Feature of Chronic Colitis

**Figure 2:**
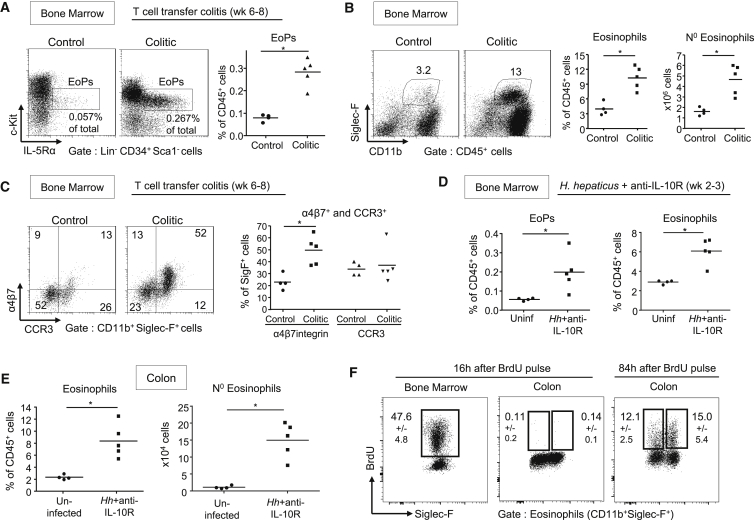
Increased Eosinopoiesis in the Bone Marrow of Colitic Mice (A–C) Colitis was induced by transfer of naive T cells into C57BL/6.*Rag1*^*−/−*^ mice. Mice were sacrificed 6 to 8 weeks after transfer (colitic) and compared to untransferred controls. (A) Representative staining and frequencies of Lin^−^CD34^+^Sca-1^−^c-Kit^int^IL-5Rα^+^ eosinophil progenitors (EoPs) among BM cells. (B) Representative staining, frequencies, and absolute numbers of eosinophils in the BM. (C) Representative staining and frequencies of α4β7 integrin and eotaxin receptor-CCR3-positive eosinophils in the BM. (D–F) Colitis was induced in C57BL/6 WT mice by infection with *H.hepaticus* (*Hh*) combined with anti-IL-10R treatment for 2 to 3 weeks (*Hh*+anti-IL-10R), which were compared to a group of uninfected and untreated mice (“uninfected,” no anti-IL10R treatment). (D) Frequencies of eosinophil EoPs and eosinophils among BM cells. (E) Frequencies and absolute numbers of eosinophils among cLPL. (F) Representative staining of BrdU incorporation by eosinophil in the BM and colon at 16 and 84 hr after initial BrdU pulsing. Frequencies ± SD are indicated (n ≥ 4). Data are representative of two independent experiments. See also Figure S2.

As we observed sustained accumulation of eosinophils in chronic intestinal inflammation, we next sought to investigate the role of eosinopoiesis in this process. Eosinophils can increase their lifespan from a few days to a few weeks within inflamed tissues (Geering et al., 2013), therefore we could not exclude that the abundant stock of preformed eosinophils in the BM (Figure 2B) fuelled tissue accumulation without major changes in eosinopoiesis. We first examined this in T cell transfer colitis and found that a striking 3.5-fold expansion of EoPs correlated with a substantial increase of eosinophils in the BM of colitic mice compared to controls (Figures 2A and 2B). There was also a 2-fold increase in the percentage of BM eosinophils positive for the gut-homing α4β7 integrin (Figure 2C). Consistent with these results, ∼7% of CD4^+^ T cells in the inflamed intestine expressed the eosinopoietin IL-5 (Figure S2A). GM-CSF, which can act in synergy with IL-5 to stimulate eosinopoiesis (Tomonaga et al., 1986), was also increased in the inflamed colon compared to controls (Figure S2B) and ∼40% of CD4^+^ T cells were GM-CSF^+^ in colitis (Figure S2A).

Next we investigated eosinopoiesis in a lymphocyte replete model of colitis to ensure our results were not a consequence of altered myelopoiesis in *Rag1*^*−/−*^ hosts. For this we used a well-described model of colitis following *Helicobacter hepaticus* (*Hh*) infection and concomitant blockade of the IL-10-IL-10R pathway (Kullberg et al., 2006). In this model, there was a similar increase in EoPs and BM eosinophils, as well as accumulation of activated TNF-secreting eosinophils in the colon (Figures 2D, 2E, and S2D).

Mature granulocytes in peripheral tissue are described as post-mitotic (Geering et al., 2013) and indeed eosinophils that accumulated in the inflamed colon stained negative for BrdU after a 16 hr pulse-chase assay, whereas almost half of eosinophils developing in the BM had incorporated the dye at this time (Figure 2F). In contrast, BrdU^+^ eosinophils appeared in the intestine only 2–3 days after initial BrdU pulsing (Figure 2F), suggesting that the increase in colonic eosinophils is supported by sustained BM eosinopoiesis.

Thus, the large accumulation of eosinophils in the inflamed intestine was supported by a significant increase in eosinopoiesis, giving rise in the BM to newly formed eosinophils that were preferentially tagged for intestinal migration.

### Eosinophilia in Colitis Is Mediated by GM-CSF-R-β Signaling

**Figure 3:**
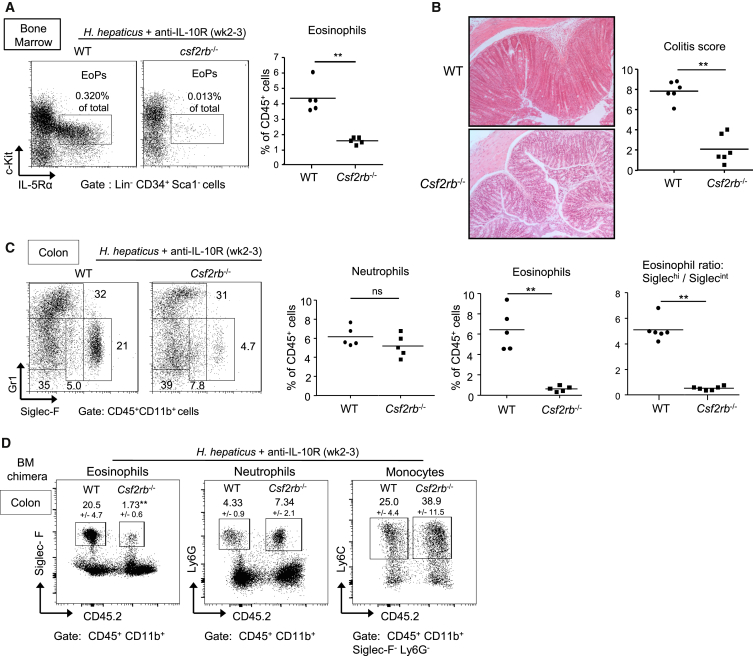
A GM-CSFR-β Chain Signal Drives Eosinophil But Not Neutrophil Accumulation and Is Required for the Development of Colitis Colitis was induced in C57BL/6 WT mice and *Csf2rb*^−/−^ mice (A–C) or mixed BM chimeric mice (D) upon infection with *Hh* combined with anti-IL-10R treatment. Mice were analyzed 2–3 weeks following induction of colitis (A–D). (A) Representative staining of EoPs and frequencies of eosinophils among total BM cells. (B) Representative photomicrographs of mid-colon sections (magnification 100×) and colitis scores. (C) Representative staining and frequencies of neutrophils and eosinophils among total cLPL and ratio of activated/resting eosinophils. Data points represent individual mice and bars represent means (A–C). (D) Representative staining of cLPL from a mixed BM chimera (WT CD45.1 and *Csf2rb*^−/−^ CD45.2). Frequencies ± SD (n = 6), gating and statistics are indicated. Data are representative of at least two independent experiments. See also Figure S3.

GM-CSF can directly stimulate eosinopoiesis and eosinophil survival (Geering et al., 2013; Tomonaga et al., 1986). To test whether the GM-CSF-eosinophil pathway is pathological in colitis in a lymphocyte-replete setting, we turned to the *Hh* and anti-IL-10R colitis model described above. Lack of a GM-CSF-Rβ signal in *Csf2rb*^−/−^ mice reduced EoP and eosinophil increases in the BM in this chronic model of colitis (Figure 3A). A change in eosinopoiesis was accompanied by a ∼90% decrease in percentages of intestinal eosinophils in *Csf2rb*^−/−^ versus WT infected mice, whereas percentages of neutrophils and CD4^+^ T cells were similar (Figures 3C and S2C). This alteration in the composition of the cellular infiltrate correlated with a significantly reduced colitis score in *Csf2rb*^−/−^ compared to WT mice (Figure 3B) and a decrease in the ratio of activated to resting eosinophils in the intestine (Figure 3C). It was notable that the decrease in intestinal eosinophils in *Csf2rb*^−/−^ mice occurred at steady state, whereas the lack of IL-23R signaling had no effect on the accumulation of eosinophils in the normal intestine (Figure S3A). Next we utilized mixed WT and *Csf2rb*^−/−^ BM chimeras to distinguish cell-intrinsic from non-cell-autonomous secondary effects. While cell-intrinsic GM-CSF-Rβ signaling was not required for accumulation of neutrophils and monocytes in the inflamed intestine, cell autonomous GM-CSF-Rβ signaling was required for eosinophil accumulation in colitis (Figure 3D).

Together, these data indicate that GM-CSF-Rβ chain signaling promotes eosinophilia and colitis and differentially regulates the accumulation of neutrophils and eosinophils in the inflamed intestine.

### Eosinophil Depletion but Not Neutrophil Depletion Ameliorates Colitis

**Figure 4:**
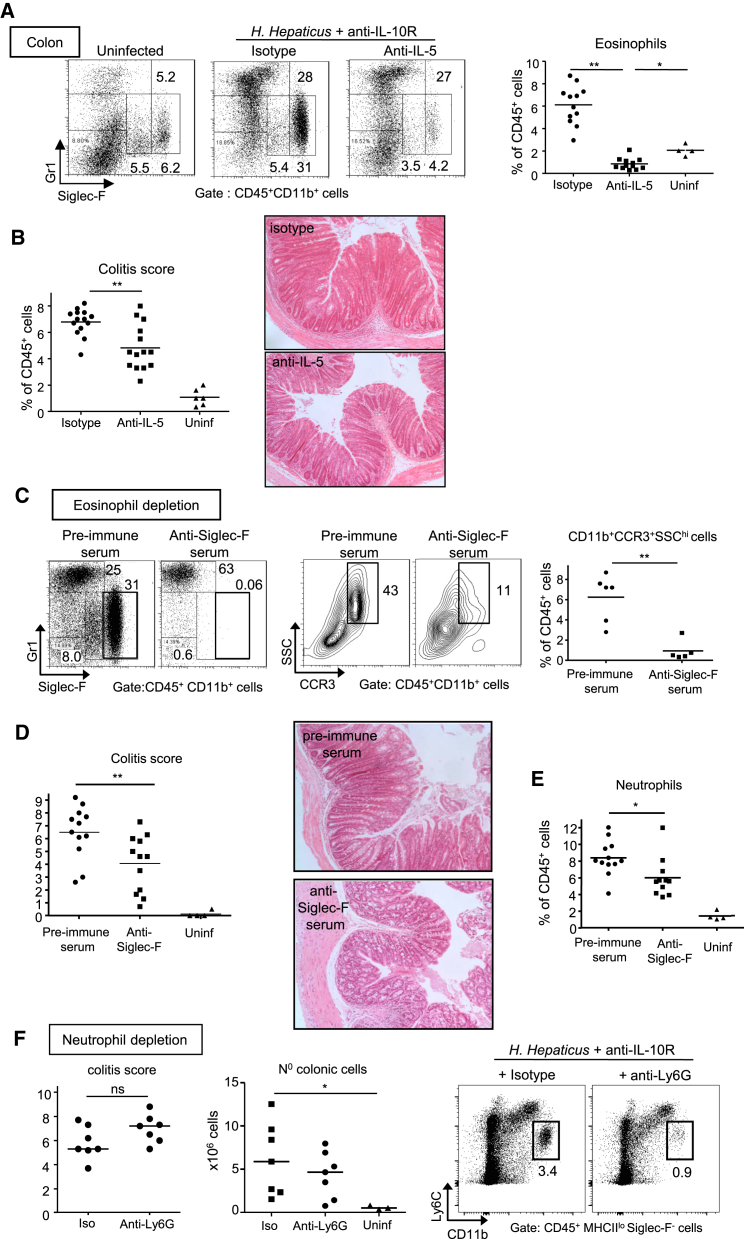
Eosinophil Depletion But Not Neutrophil Depletion Ameliorates Colitis WT mice were infected with *Hh* combined with anti-IL-10R treatment and analyzed 2–3 weeks later. Mice received either 2 weekly injections of anti-IL-5 or isotype control mAbs (A and B), or sheep anti-Siglec-F serum or pre-immune serum (C–E) for eosinophil depletion, or 3 weekly injections of anti-Ly6G or isotype control for neutrophil depletion (F). Infected mice were compared to uninfected and untreated controls (“uninf”). (A) Representative staining and frequencies of eosinophils among cLPL. (B) Colitis score and representative photomicrographs of mid-colon sections (magnification 100x). (C) FACS staining of SiglecF^+^ eosinophils (left) and SSC^hi^ CCR3^+^ eosinophils (middle) and frequencies of CD11b^+^CCR3^+^SSC^hi^ eosinophils among cLPL (right). (D) Colitis score and representative photomicrographs of mid-colon sections (magnification 100x). (E) Frequencies of neutrophils among cLPL. (F) Colitis score (left), total numbers of cLPL (middle) and representative staining of neutrophils among cLPL (right). Data are pooled from (A–E) or are representative of (F) two independent experiments. See also Figure S4.

Because the lack of colitis observed in *Csf2rb*^−/−^ mice correlated with a decrease in the frequency of eosinophils, but not neutrophils, we next investigated the relative contribution of these distinct innate effectors to the pathogenesis of colitis. We employed two strategies to deplete eosinophils, either blockade of the eosinopoietin IL-5 (Kouro and Takatsu, 2009) or antibody-mediated depletion of Siglec-F^+^ cells. In the presence of IL-10R blockade, *Hh* infected mice treated with anti-IL-5 had a 50% reduction in total cLPL compared to isotype treated controls (data not shown) and an 87% decrease in the percentage of Siglec-F^hi^ eosinophils (Figure 4A). Most importantly, this was accompanied by a significant reduction in colitis severity compared to isotype treated controls (Figure 4B).

The IL-5R is constitutively expressed by eosinophils but also by some B cell subsets (Kouro and Takatsu, 2009). Therefore, to further increase the specificity of treatment, we used an anti-Siglec-F depletion approach shown to selectively eliminate eosinophils (Chu et al., 2014; Zimmermann et al., 2008). Although alveolar macrophages in the lung express Siglec-F, intestinal and peritoneal macrophages do not and are therefore not affected by anti-Siglec-F depletion (Feng and Mao, 2012; Mowat and Bain, 2011). Treatment with anti-mouse Siglec-F immune serum reduced colitis severity to the same extent as IL-5 blockade (Figure 4D). This treatment regimen led to an 85% decrease of eosinophils based on reduced CD11b^+^CCR3^+^SSC^hi^ cells in the colon of anti-Siglec-F versus pre-immune serum treated mice (Figure 4C), whereas there was only a 28% reduction in the percentage of colonic neutrophils (Figure 4E). The small reduction in neutrophils was most likely secondary to reduced overall inflammation, because uninfected mice treated with anti-Siglec-F serum did not display a decrease in neutrophils or any leukocyte populations other than eosinophils (Figure S4A). By contrast with eosinophil-depleting strategies, depletion of neutrophils with an anti-Ly-6G antibody did not have a significant effect on colitis (Figures 4F and S4B).

Together these results reveal differential roles for eosinophils and neutrophils in chronic colitis. While eosinophils play a non-redundant role in disease, neutrophils are dispensable for the development of chronic intestinal inflammation.

### GM-CSF Sustains the Accumulation of Eosinophils with an Activated Phenotype in Colitis

**Figure 5:**
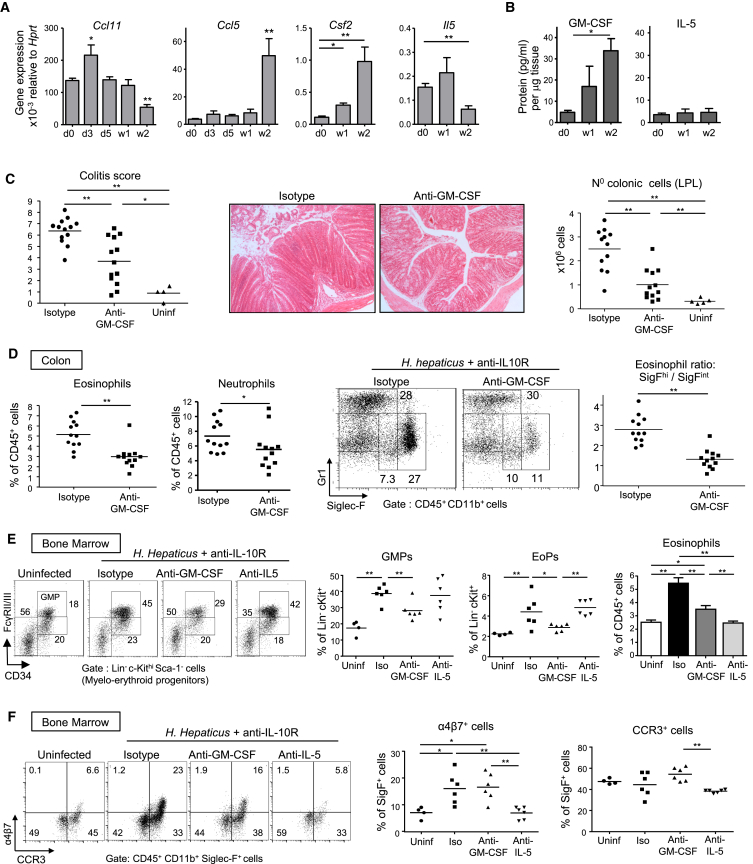
Specific Blockade of GM-CSF Decreases Colitis and Eosinophilia WT mice were infected with *Hh* combined with anti-IL-10R treatment. (A) qPCR gene expression from total colonic tissue. (B) Cytokine expression of colonic explant supernatants cultured for 24 hr and normalized to tissue weight (n ≥ 5 per group). (C–F) Mice received two weekly injections of anti-GM-CSF or isotype control mAbs and were analyzed after 2–3 weeks along with unmanipulated controls (uninf). (C) Colitis score, representative photomicrographs of mid-colon sections (magnification 100×) and absolute numbers of total cLPL. (D) Frequencies of Siglec-F^hi^ eosinophils and neutrophils among CD45^+^ cLPL (left), representative FACS staining (middle) and ratio of activated/resting colonic eosinophils (right). (E) Representative staining and frequencies of BM granulocyte-monocyte precursors (GMPs), frequencies of EoPs among Lin^−^Sca-1^−^c-Kit^hi^ cells and of eosinophils among BM cells. (F) Representative staining and frequencies of α4β7^+^ eosinophils and CCR3^+^ eosinophils in the BM. Data are pooled from (A–D) or are representative of (E and F) two independent experiments. Error bars represent SD. See also Figure S5.

In order to further understand the colitogenic role of GM-CSF, we investigated whether GM-CSF and IL-5 had differential effects on eosinophil production and activation. Because GM-CSF-R and IL-5R share the same β-receptor subunit (Kouro and Takatsu, 2009), we tested whether GM-CSF blockade would reproduce the decrease in eosinophil activation and accumulation observed in *Csf2rb*^−/−^ mice. Interestingly, IL-5 and GM-CSF were produced at steady state by ILCs but were not increased in lymphocyte-replete colitis (Figure S5B). By contrast, GM-CSF production by CD4^+^ T cells was increased in the inflamed colon compared to controls, while percentages of IL-5 producers were unchanged (Figure S5A). Accordingly, colonic GM-CSF, but not IL-5, mRNA and protein levels were augmented in chronic colitis, possibly highlighting a more homeostatic role for IL-5 compared with the more activation-induced functions of GM-CSF (Figure 5A and 5B). Regarding eosinophil chemoattractants, eotaxin-1 and RANTES were increased in early and late phases of colitis, respectively (Figure 5A).

When treated with anti-GM-CSF, *Hh* infected and anti-IL-10R-treated mice exhibited significantly reduced colonic infiltrates and colitis score compared to mice treated with isotype control (Figure 5C) and displayed a striking 50% decrease in the frequency and activation status of colonic eosinophils (Figure 5D). While anti-IL-5 treatment inhibited the general accumulation of colonic eosinophils (Siglec-F^int^ and Siglec-F^hi^) (Figures 4A and S5D), GM-CSF blockade only decreased the most activated population (Figures 5D and S5D) suggesting a role for GM-CSF in intestinal eosinophil activation in the inflamed colon.

Blockade of either GM-CSF or IL-5 led to reductions in the number of eosinophils in the BM; however, only GM-CSF blockade inhibited the accumulation of GMPs and downstream EoPs (Figure 5E). These results indicate that during intestinal inflammation, GM-CSF sustains eosinophilic granulopoiesis, whereas IL-5 mediates a more specific function promoting the terminal differentiation of EoPs into Siglec-F^+^ cells (Figure 5E). A differential effect of GM-CSF and IL-5 was also evident on the “gut-tagging” of newly produced BM eosinophils, as upregulation of α4β7 integrin in colitis was only inhibited by IL-5 blockade (Figure 5F).

Overall, these results highlight the synergy between GM-CSF and IL-5 in the regulation of eosinopoiesis and reveal the key role of GM-CSF in driving chronic intestinal inflammation through accumulation of activated eosinophils in the colon.

### GM-CSF Promotes the Effector Functions of Mature Eosinophils in the Inflamed Intestine

**Figure 6:**
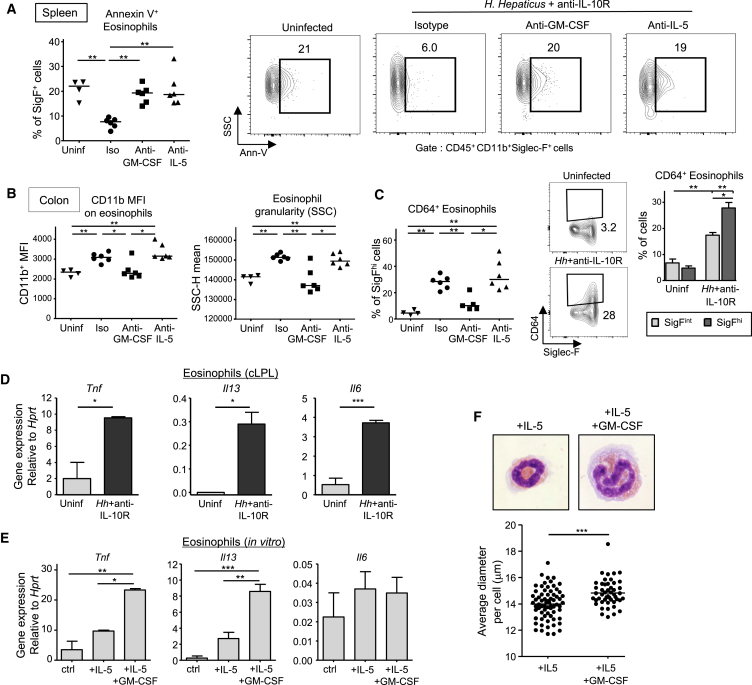
GM-CSF Promotes Colonic Eosinophil Activation and Effector Functions (A–D) WT mice were infected with *Hh* combined with anti-IL-10R treatment and analyzed after 2–3 weeks along with unmanipulated controls (i.e., uninfected and untreated: “Uninf”). Where indicated, mice received two weekly injections of anti-GM-CSF, anti-IL-5, or isotype control mAbs. (A) Frequencies and representative staining of apoptotic Annexin V^+^ (AnnV) eosinophils among splenocytes. (B) CD11b and SSC MFI of colonic eosinophils. (C) Representative staining and percentages of CD64^+^ cells among colonic eosinophils. (D) Cytokine genes expression profile assessed by qPCR from FACS-sorted eosinophils isolated from the colon of unmanipulated mice (Uninf) or colitic mice (*Hh*+anti-IL-10R). (E and F) FACS-sorted eosinophils isolated from the colon of unmanipulated mice were stimulated for 18 hr with IL-5 or a combination of IL-5 and GM-CSF or were left untreated (ctrl). (E) qPCR analysis of cytokine genes expression after in vitro stimulation. (F) Representative Diff-Quick staining (top) and cell size analysis (bottom) of eosinophil cytospins after in vitro stimulation. Dots represent individual eosinophils and bars indicate means. Data are representative of at least two experiments. Error bars represent SD.

We next sought to characterize further the differential regulation of eosinophils in the periphery by GM-CSF and IL-5. Both cytokines can promote eosinophil survival (Geering et al., 2013), and we confirmed this observation in colitis. Annexin-V staining on freshly isolated peripheral eosinophils was decreased in colitis and increased in the presence of GM-CSF or IL-5 blockade (Figure 6A). As GM-CSF production was increased during colitis, while IL-5 levels stayed constant (Figure 5B), we hypothesized that GM-CSF would be a key driver of eosinophil effector functions in the inflamed intestine. Indeed, anti-GM-CSF treatment inhibited the increase in CD11b and increase in side scatter (correlating with granularity), while IL-5 blockade did not have a significant effect on these markers of activation (Figure 6B). In addition, cell-sorted eosinophils exhibited morphological changes in vitro in the presence of GM-CSF, notably increased diameter as a sign of activation (Figure 6F). Interestingly CD64 (FcγRI), which is increased on neutrophils in IBD (Minar et al., 2014), was induced on eosinophils during colitis in a GM-CSF-dependent but IL-5-independent manner (Figure 6C). Furthermore, the amount of CD64 was higher on Siglec-F^hi^ than Siglec-F^int^ eosinophils (Figure 6C), consistent with their more activated status.

Regarding expression of cytokines involved in epithelial cell dysregulation and damage (Neurath, 2014), intestinal eosinophils expressed higher amounts of *Tnf*, *Il6*, and *Il13* mRNA in colitis compared to uninflamed controls (Figure 6D). In vitro analysis of cell-sorted intestinal eosinophils showed that GM-CSF stimulated *Tnf* and *Il13* mRNA expression, but had no effect on *Il6* (Figure 6E). Altogether, these data demonstrate that GM-CSF and IL-5 promote the survival of peripheral eosinophils, but only GM-CSF promotes their activation and inflammatory cytokine production, revealing one of the key colitogenic effects of GM-CSF during chronic intestinal inflammation.

### Eosinophil Peroxidase Activity Promotes Chronic Colitis

**Figure 7:**
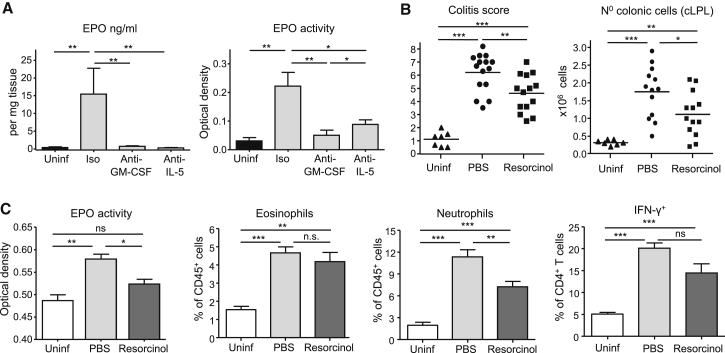
Eosinophil Peroxidase Activity Promotes Intestinal Inflammation WT mice were infected with *Hh* combined with anti-IL-10R administration and were either treated with two weekly injections of anti-GM-CSF, anti-IL-5, or isotype control mAbs (A) or injected daily with Resorcinol or PBS (B and C). Mice were analyzed 2–3 weeks after induction of colitis and compared to uninfected and untreated controls (Uninf). (A) ELISA of eosinophil peroxidase (EPO) (left) or colorimetric assay of EPO activity (middle) assessed in the supernatants of colonic explants cultured for 24 hr and normalized to tissue weight (n ≥ 6 per group). (B) Colitis score and absolute numbers of cLPL. (C) Colorimetric assay of EPO activity assessed on the supernatants of total colonic faeces (n = 5–7, left), frequencies of eosinophils and neutrophils among total cLPL (middle), and percentages of IFN-γ^+^ cells among colonic CD4^+^ T cells (right). Data are pooled from two independent experiments. Error bars represent SD.

Because TNF, IL-6, and IL-13 are expressed by various leukocytes, we decided to investigate whether eosinophil-specific products could also drive chronic intestinal inflammation. For this purpose, we tested whether EPO, which is produced exclusively by eosinophils and can be tissue-toxic (Fulkerson and Rothenberg, 2013), contributed to chronic colitis. EPO levels and activity in the intestine were greatly increased during chronic inflammation, confirming substantial eosinophil degranulation. In addition, a reduction in eosinophil numbers during anti-GM-CSF or anti-IL-5 treatment was accompanied by a significant decrease in EPO (Figure 7A). Resorcinol is a potent inhibitor of EPO leading to decreased anti-bacterial activity of eosinophils (Ledford et al., 2012). In *Hh*-induced colitis, daily treatment of WT mice with resorcinol led to significantly reduced EPO activity and decreased colitis (Figures 7B and 7C). This was accompanied by decreased markers of colonic inflammation compared to PBS treated mice, including reduced leukocyte infiltration, lower neutrophil percentages, and a trend toward reduced IFN*-*γ^+^ CD4^+^ T cells (Figures 7B and 7C). EPO inhibition, however, did not affect the frequency of eosinophils among cLPL (Figure 7C), consistent with previous in vivo observations (Forbes et al., 2004; Ledford et al., 2012).

Overall, the pathogenic effect of uncontrolled accumulation of activated eosinophils in chronic colitis could be attenuated by inhibition of EPO, an enzyme well known to mediate oxidative tissue damage in eosinophil-dependent inflammatory diseases (Fulkerson and Rothenberg, 2013).

## Discussion

Our study newly identifies a GM-CSF-eosinophil axis as a crucial component of IL-23-driven chronic colitis. Our previous work described GM-CSF as a pivotal downstream effector of IL-23 in the inflammatory cascade that drives aberrant responses to commensal microbiota through increases in myelopoiesis in T cell transfer colitis (Griseri et al., 2012). Neutrophils are widely accepted as tissue-toxic cells in IL-23-mediated colitis (Chin and Parkos, 2006; Neurath, 2014). However, our results challenge this view and indicate a more prominent and unexpected role for eosinophils in this response. We show a marked accumulation of activated eosinophils in the colon of colitic mice, supported by increased eosinopoiesis, and a direct colitogenic role through production of eosinophil peroxidase and inflammatory cytokines. Although eosinophils are abundant in the intestine, their role in chronic intestinal inflammation is rarely considered. Here we identified GM-CSF as a key molecular switch diverting eosinophils from a tissue-protective to a tissue-toxic state of activation. These results extend the paradigm of eosinophil-mediated immune pathology beyond Th2 cell-type responses to effectors of IL-23-GM-CSF-driven dysregulated tissue immunity.

GM-CSF is emerging as a central cytokine at the crossroads of various types of effector T cell responses and can be produced by Th1 and Th2 cells to stimulate increased myeloid cell activity (Mosmann and Coffman, 1989). More recently it was shown that IL-23 stimulated Th17 cells to produce GM-CSF, which was pathogenic in EAE although its functional role was not established (El-Behi et al., 2011). In the inflamed intestine, IL-23 stimulated polyfunctional IFNγ^+^IL-17A^+^Th cells to produce GM-CSF, which triggered extramedullary hematopoiesis (Griseri et al., 2012). In this report, we show that GM-CSF increased eosinopoiesis and numbers of highly activated eosinophils in the inflamed intestine. GM-CSF promoted increases in GMPs and downstream EoPs, which both express GM-CSF-R-α and β chains (Iwasaki et al., 2005). Increased EoPs have been observed in Th2 cell-mediated asthma and anti-helminth responses (Iwasaki et al., 2005; Yang et al., 2011), however, our study constitutes the first report of chronic EoP accumulation during IL-23-Th17 cell type-mediated immune disease, extending our previous observation of dysregulated hematopoiesis in colitis to the eosinophilic lineage.

IL-5 or GM-CSF blockade resulted in a substantial decrease of eosinophils in the inflamed intestine, however there were marked differences in their action. Although both cytokines promoted eosinopoiesis during colitis, IL-5 specifically increased the differentiation of EoPs into Siglec-F^+^ eosinophils and promoted imprinting of α4β7 integrin expression. However by contrast with GM-CSF it had no effect on the upstream GMP.

Resident populations of intestinal leukocytes contribute to the maintenance of basal eosinophil numbers as ILCs constitutively produce IL-5 in the normal intestine and resident macrophages express eotaxins (Ahrens et al., 2008; Nussbaum et al., 2013). However, intestinal IL-5 does not appear to be controlled by IL-23 because IL-5 expression was not increased in colitis or reduced in IL-23 deficiency. These results are in contrast to the findings that IL-23 promoted IL-5 and Th2 responses in asthma models suggesting differences in IL-5 regulation in distinct tissue sites (Peng et al., 2010; Wakashin et al., 2008). In contrast with IL-5, IL-23 increased GM-CSF expression by CD4^+^ T cells and ILC in colitis (Griseri et al., 2012 and data not shown), pinpointing an IL-23-GM-CSF-eosinophil axis in colitis that can boost basal IL-5 dependent eosinophilia. A recent study showed a role for eosinophils in maintaining intestinal integrity toward the gut microbiota through stimulating IgA^+^ plasma cells and Foxp3^+^ Treg cells (Chu et al., 2014). Importantly, here we show that the unchecked production of GM-CSF during chronic colitis is a key driver of the eosinophil switch from a resident and homeostatic phenotype (Siglec-F^int^) to an over-activated and tissue toxic phenotype (Siglec-F^hi^). Siglec-F^hi^ eosinophils were also increased in lung inflammation and were more resistant to apoptosis than Siglec-F^int^ eosinophils (Ohnmacht et al., 2007). Eosinophil activation and cytokine secretion that accompanied colitis was inhibited by GM-CSF but not IL-5 blockade. Furthermore in vitro, GM-CSF acted directly on eosinophils to induce production of colitogenic cytokines TNF and IL-13. Together the data suggest that IL-5 plays a homeostatic role maintaining basal levels of eosinophils in the intestine, whereas GM-CSF promotes their activation and deleterious effector functions in chronic colitis.

It is worth noting that GM-CSF-Rβ deficiency did not affect the percentage of neutrophils in the intestine in colitis and BM chimera experiments, despite inducing a severe decrease in eosinophil percentages. Thus, GM-CSF is not absolutely required for the neutrophil increase probably owing to the compensatory role of G-CSF, which is a potent inducer of neutrophilia (Nathan, 2006) and is increased in T cell transfer colitis (Griseri et al., 2012). Unexpectedly, while eosinophil depletion dampened colitis, no such effect was provoked by depletion of neutrophils, highlighting a dichotomy in the role of these granulocyte populations in chronic colitis.

Amelioration of chronic colitis by pharmacological inhibition of EPO, which is implicated in cytotoxic oxidant generation, pinpointed one of the molecular mechanisms by which eosinophils specifically mediate intestinal damage. This pathway has also been implicated in the DSS model of acute colonic injury (Forbes et al., 2004), suggesting broad relevance in intestinal damage. Interestingly, regulation of eosinophils in acute versus chronic intestinal inflammation is not identical as we found that IL-5 depletion inhibited chronic colitis, whereas IL-5 deficiency had no significant effect in the DSS model contrary to the protective effect of eotaxin deficiency (Forbes et al., 2004). This suggests that in an acute damage model, mobilization of mature eosinophils from the BM to intestine is sufficient, whereas sustained chronic colitis requires IL-5-dependent eosinopoiesis.

In human, treatment of eosinophils with GM-CSF in vitro led to increased release of EPO and ECP, providing further evidence that GM-CSF can directly increase the cytotoxic functions of eosinophils (Pazdrak et al., 2011). Conversely, IL-10 inhibited LPS-induced TNF release and increased survival of human eosinophils in vitro (Takanaski et al., 1994). Treg cells play an important role in intestinal homeostasis and suppress colitis in part via IL-10 (Maloy and Powrie, 2011). We found that Treg cell-mediated control of colitis correlated with a reduction in eosinophil accumulation and activation. Based on those results, it is tempting to speculate that under homeostatic conditions eosinophils in the intestine are hyporesponsive to TLR activation as a consequence of the IL-10 rich environment (Maloy and Powrie, 2011), which might be over-ridden by sustained increases in GM-CSF production in chronic inflammation.

There are several reports of increased GM-CSF in Crohn’s disease (CD) and ulcerative colitis (UC) patients (Ina et al., 1999; Noguchi et al., 2001), and concomitant Th17 and IL-5 and IL-13 T cell responses have been observed in ileal CD suggesting a more polyfunctional T cell response in certain patients (Zorzi et al., 2013). However GM-CSF activity is a double-edged sword and when produced in a controlled fashion plays an important role in the steady state accumulation of mononuclear phagocytes and Foxp3^+^ Treg cells (Mortha et al., 2014) and in promoting host protective immunity in the gut (Hirata et al., 2010). Consistent with this, intestinal injury in a DSS model was exacerbated in *Csf2rb*^−/−^ mice further illustrating differences in the mechanisms of acute and chronic intestinal damage (Egea et al., 2013). Increased levels of anti-GM-CSF autoantibodies have been observed in pediatric and some forms of adult CD leading to the idea that GM-CSF is protective in IBD. However three clinical trials of recombinant GM-CSF administration failed to show demonstrable protective effects (Roth et al., 2012). It is highly likely that GM-CSF will play both protective and pathological roles in IBD and that the context in which it is produced, such as where and for how long, might determine its ultimate functional role.

Several studies have reported increased eosinophil numbers and activation in both UC and CD (Ahrens et al., 2008; Saitoh et al., 1999). ECP was also increased in the faeces of IBD patients suggesting eosinophil degranulation (Bischoff et al., 1997). Our results in model systems taken together with an emerging picture in humans suggest that blockade of the GM-CSF/eosinophil axis might be a therapeutic target in particular patient subsets. The fact that sustained depletion of eosinophils in patients with hyper-eosinophilic syndrome treated for up to 6 years with anti-IL-5 did not lead to adverse effects is encouraging for considering this approach in IBD (Gleich et al., 2013).

## Experimental Procedures

### Mice

B6.SJL-Cd45.1 mice or C57BL/6 mice: wild-type (WT), *Csf2rb*^−/−^, *Il23r*^*−/−*^, *Rag1*^*−/−*^, or *Rag1*^*−/−*^*Il23p19*^*−/−*^ were bred and maintained under specific pathogen–free conditions in accredited animal facilities at the University of Oxford. All procedures involving animals were conducted according to the requirements and with the approval of the UK Home Office Animals (Scientific Procedures) Acts, 1986. Mice were negative for *Helicobacter* spp. and other known intestinal pathogens and were used when 7–12 weeks old.

### Induction of T Cell Transfer Colitis

Naive CD4^+^CD25^−^CD45RB^hi^ T cells and regulatory CD4^+^CD25^+^CD45RB^lo^ T cells were sorted by flow cytometry from enriched CD4^+^ single-cell spleen suspensions (Dynal) to a purity of >99%. For the induction of colitis, 4 × 10^5^ naive T cells were injected intraperitoneally (i.p.) into *C57BL/6.Rag1*^−/−^ recipients. Where indicated, 2 × 10^5^ protective T reg cells were co-injected i.p.

### *Helicobacter hepaticus*-Dependent Induction of Colitis

Colitis was induced in WT C57BL/6 mice by infecting with *Hh* (oral gavage) on 2 consecutive days with 5 × 10^7^–2 × 10^8^ CFU *Hh* and i.p. injection of 1 mg 1B1.2 (anti-IL10R) mAb on days 0 and 7 after *Hh* infection (Buonocore et al., 2010). Mice were killed 7 days after the last anti-IL10R mAb treatment (weeks 2–3). Where indicated, mice were i.p. injected two times per week with 0.4 mg of anti-GM-CSF (MP1-22E9; CSL Ltd) or isotype control (GL117, rat IgG2a) or 0.5 mg of anti-IL-5 (TRFK5; BioXCell), or three times per week with 0.25 mg anti-Ly6G (1A8; BioXCell) or isotype control (2A3, rat IgG2a) starting from the first day of *Hh* infection. Where indicated, mice were i.p. injected two times per week with sheep preimmune serum or sheep anti-Siglec-F serum. Where indicated, mice were i.p. injected daily with Resorcinol (1.25mg/kg) or PBS.

### Histological Assessment of Intestinal Inflammation

Proximal, mid-, and distal colon samples were fixed in buffered 10% formalin solution. 5 μm paraffin embedded sections were cut and stained with hematoxylin and eosin and inflammation was scored in a blinded fashion (Buonocore et al., 2010). In brief, 4 parameters of inflammation were assessed (scored 0–3): epithelial hyperplasia and goblet cell depletion, leukocyte infiltration in the lamina propria, area of tissue affected, and markers of severe inflammation such as submucosal inflammation. Aggregate scores were taken for each section, to give a total inflammation of 0–12. Colon inflammation scores represent the average score of the three sections taken.

### Leukocytes Isolation

Single cell suspensions were prepared from spleen, MLN, and cLPL as previously described (Buonocore et al., 2010). In brief, colons were longitudinally opened, cut into 1 cm pieces, and incubated in RPMI 1640 with 10% FCS and 5 mM EDTA at 37°C to remove epithelial cells. Tissue was then digested with 100 U/ml of type VIII collagenase (Sigma) in complete RPMI medium containing 15cmM HEPES during 1 hr at 37°C. The isolated cells were layered on a 30/40/75% Percoll gradient, which was centrifuged for 20 min at 600 *g*, and the 40/75% interface, containing mostly leukocytes, was recovered. BM cell suspensions were prepared by flushing the marrow out of femur and tibia and were resuspended in PBS with 2% BSA.

Flow cytometry and cell sorting, quantitation of gene expression using real-time PCR, in vitro stimulation assays, EPO Elisa and EPO colorimetric assay, in vivo BrdU labeling and immunofluorescence were performed as described in Supplemental Experimental Procedures.

### Statistical Analysis

Statistical analysis was performed with Prism 6.0 (GraphPad Software). The nonparametric Mann-Whitney test was used for all statistical comparisons. Differences were considered statistically significant when p < 0.05.

## Author Contribution

T.G. and I.C.A planned and performed experiments and wrote the paper. C.P., T.K., C.S., F.F., and J.S. performed particular experiments. F.P. wrote the paper and supervised the study. F.P. and T.G. designed the study. B.S.M. and P.R.C. provided essential materials and were involved in data discussions.

## Supplemental Information

Document S1. Figures S1–S5 and Supplemental Experimental Procedures

Document S2. Article plus Supplemental Information
