# Supplementary material for: Granulocyte Macrophage Colony-Stimulating Factor-Activated Eosinophils Promote Interleukin-23 Driven Chronic Colitis
Source: Immunity. 2015 Jul 21;43(1):187–99. doi: 10.1016/j.immuni.2015.07.008 (PMC4518500; doi:10.1016/j.immuni.2015.07.008)
Supplement: Document S1. Figures S1–S5 and Supplemental Experimental Procedures [file mmc1.pdf]

Immunity

Supplemental Information

Granulocyte Macrophage Colony-Stimulating Factor-Activated Eosinophils Promote Interleukin-23 Driven Chronic Colitis

Thibault Griseri, Isabelle C. Arnold, Claire Pearson, Thomas Krausgruber, Chris Schiering, Fanny Franchini, Julie Schulthess, Brent S. McKenzie, Paul R. Crocker, and Fiona Powrie

Figure S1

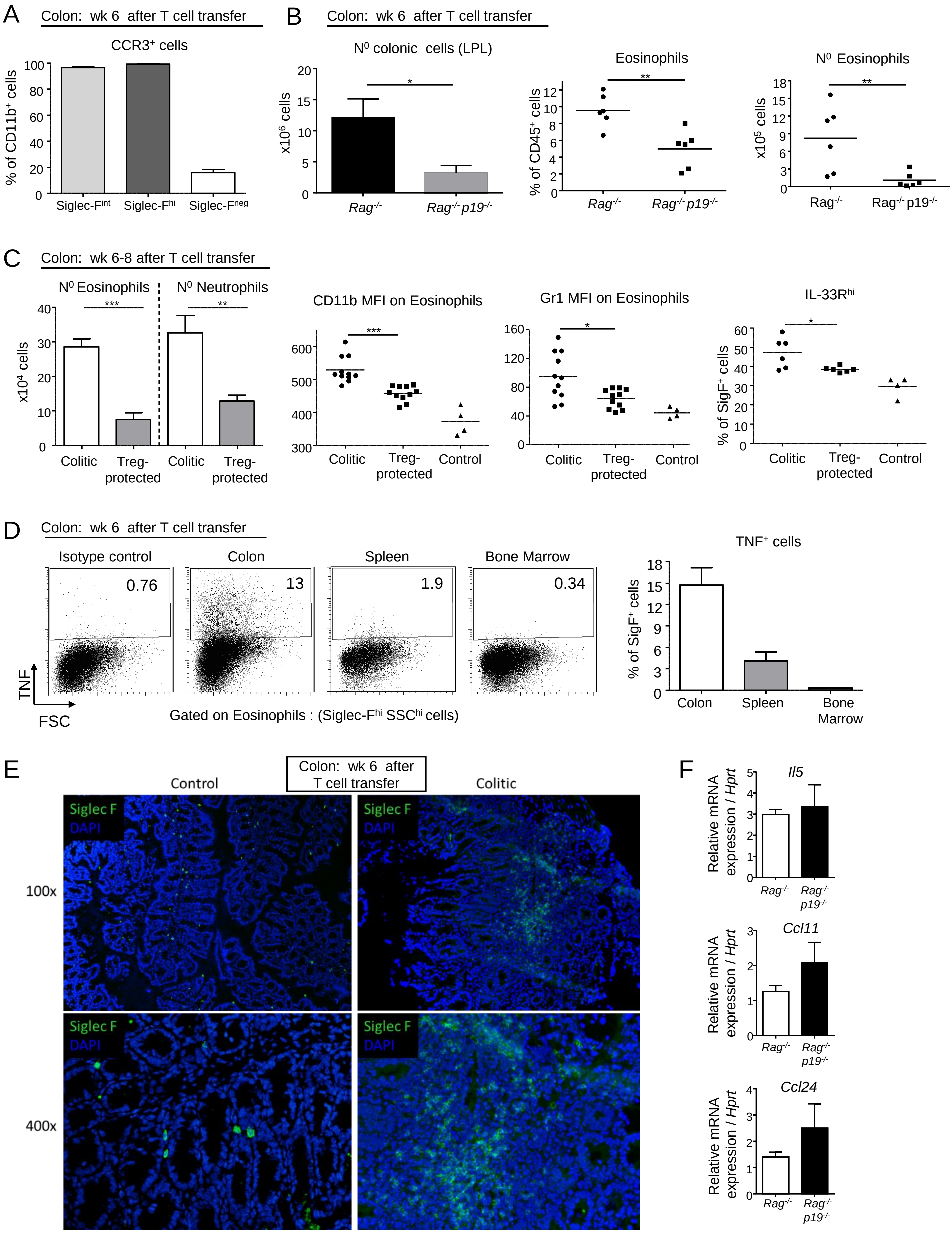

A
Colon: wk 6 after T cell transfer
CCR3+ cells
% of CD11b+ cells
Siglec-Fint
Siglec-Fhi
Siglec-Fneg
B
Colon: wk 6 after T cell transfer
N0 colonic cells (LPL)
x106 cells
Rag-/-
Rag-/- p19-/-
Eosinophils
% of CD45+ cells
N0 Eosinophils
x105 cells
C
Colon: wk 6-8 after T cell transfer
N0 Eosinophils
N0 Neutrophils
x104 cells
Colitic
Treg-protected
CD11b MFI on Eosinophils
Gr1 MFI on Eosinophils
IL-33Rhi
% of SigF+ cells
Control
D
Colon: wk 6 after T cell transfer
Isotype control
Colon
Spleen
Bone Marrow
0.76
13
1.9
0.34
TNF
FSC
Gated on Eosinophils : (Siglec-Fhi SSChi cells)
TNF+ cells
% of SigF+ cells
E
Colon: wk 6 after T cell transfer
Control
Colitic
Siglec F
DAPI
100x
400x
F
Il5
Ccl11
Ccl24
Relative mRNA expression / Hprt
Rag-/-
Rag-/- p19-/-

Figure S1 (related to Figure 1). CCR3 expression and TNF production by eosinophils; IL-23 and T reg cell mediated eosinophil regulation

Colitis was induced by transfer of naïve $CD4^{+}CD45RB^{hi}$ T cells into C57BL/6.*Rag1*$^{-/-}$ or *Rag1*$^{-/-}$*Il23p19*$^{-/-}$ mice, as indicated. Mice were analyzed 6 to 8 weeks after transfer **(A-F)**. **(A)** Frequencies of $CCR3^{+}$ cells among $CD11b^{+}$ colonic lamina leukocytes (cLPL) (± SEM, n=5 mice per group). **(B)** Total number of cLPL, frequencies and absolute numbers of Siglec-F^{hi} eosinophils. As described in (Hue et al., 2006), T cell transferred *Rag1*$^{-/-}$*Il23p19*$^{-/-}$ mice had mild colonic inflammation while *Rag1*$^{-/-}$ mice had severe colitis. **(C)** T cell transferred *Rag1*$^{-/-}$ mice (colitic) were compared to untransferred *Rag1*$^{-/-}$ mice (control) or mice co-transferred with protective $CD25^{+}$ Treg cells (Treg-protected). Absolute numbers of Siglec-F^{hi} eosinophils and neutrophils within cLPL (left). CD11b and GR1 MFI on colonic eosinophils (middle) and frequencies of IL-$33R^{hi}$ expressing eosinophils (right). **(D)** Total cLPL, splenocytes and BM cells from *Rag1*$^{-/-}$ colitic mice were restimulated for 4 hours with PMA/ionomycin/Brefeldin A to assess intracellular TNF expression. Representative FACS staining and frequencies of TNF^{+} eosinophils (± SEM, n=5 mice per group) are shown. Each point represents an individual mouse and horizontal bars represent group means. **(E)** Staining for eosinophils (Siglec-F^{+}) by immunofluorescence in the colon of untransferred controls or colitic *Rag1*$^{-/-}$ mice. The same section is represented in the four panels. **(F)** *Il5*, *Ccl11* (eotaxin 1) and *Ccl24* (eotaxin 2) gene expression profile assessed by qPCR from total colon homogenate (n=4). Values are normalized to HPRT and represent means (± SEM). **(A, C**, **D)** Data are representative of two independent experiments.

Figure S2

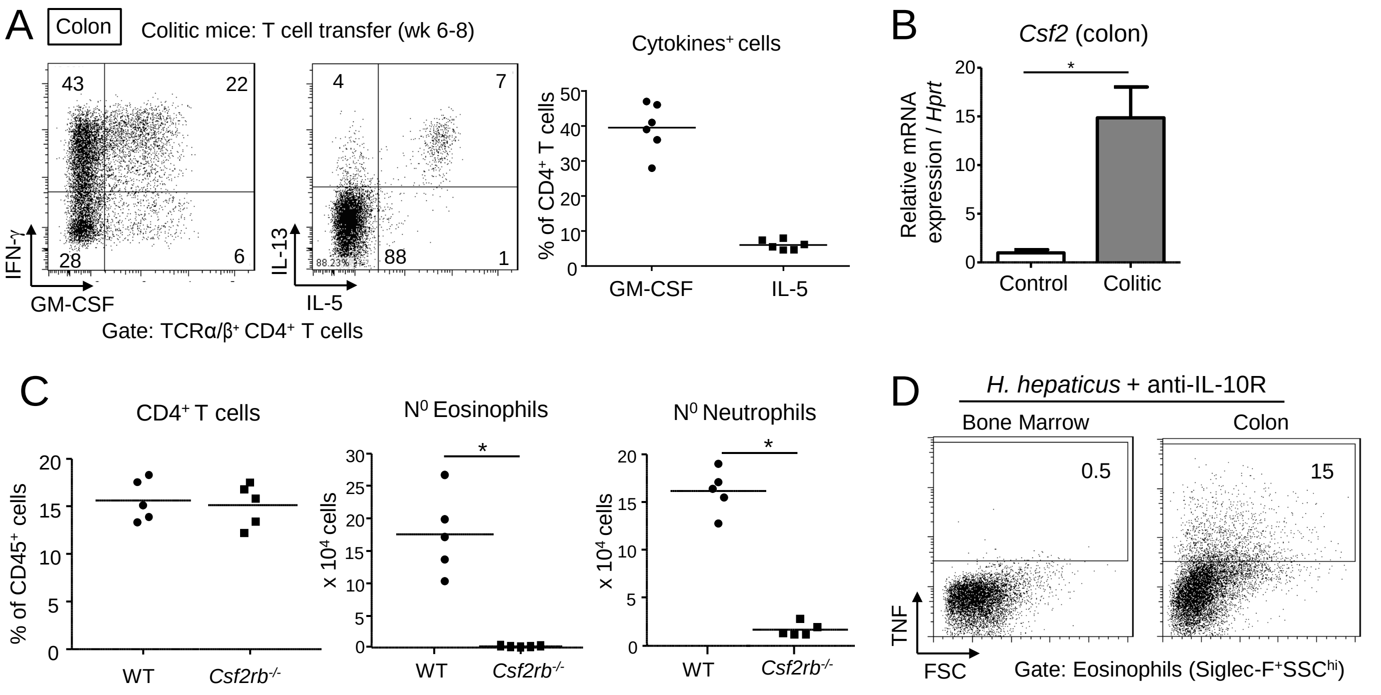

Figure S2 (related to Figure 2). GM-CSF and IL-5 expression in T cell transfer colitis

(A-B) Colitis was induced by transfer of naïve $CD4^+CD45RB^{hi}$ T cells into C57BL/6.*Rag1*$^{-/-}$ mice. T cell transferred mice (colitic) were killed after 6 wk and compared with untransferred *Rag1*$^{-/-}$ mice (control). **(A)** cLPL were restimulated 4 hours with PMA/ionomycin/Brefeldin A to assess intracellular cytokines expression. Representative FACS staining and frequencies of GM-CSF$^+$ and IL-5$^+$ cells among TCRα/β$^+$ CD4$^+$ T cells. **(B)** *Csf2* (GM-CSF) gene expression assessed by qPCR from total colon homogenate (n=4). Values are normalized to HPRT and represent means (± SEM). **(C-D)** Colitis was induced in C57BL/6 WT mice upon infection with *H.hepaticus* (*Hh*) combined with anti-IL-10R treatment. Where indicated, WT mice were compared to *Csf2rb*$^{-/-}$ mice and analysed 2-3 weeks following induction of colitis. **(C)** Frequencies of CD4$^+$ T cells and absolute numbers of eosinophils and neutrophils cells among cLPL. Each point represents individual mice and horizontal bars represent group means. **(D)** Total bone-marrow cells and cLPL were restimulated for 4 hours with PMA/ionomycin/Brefeldin A to assess intracellular TNF expression. Representative FACS staining of TNF$^+$ eosinophils is shown.

Figure S3

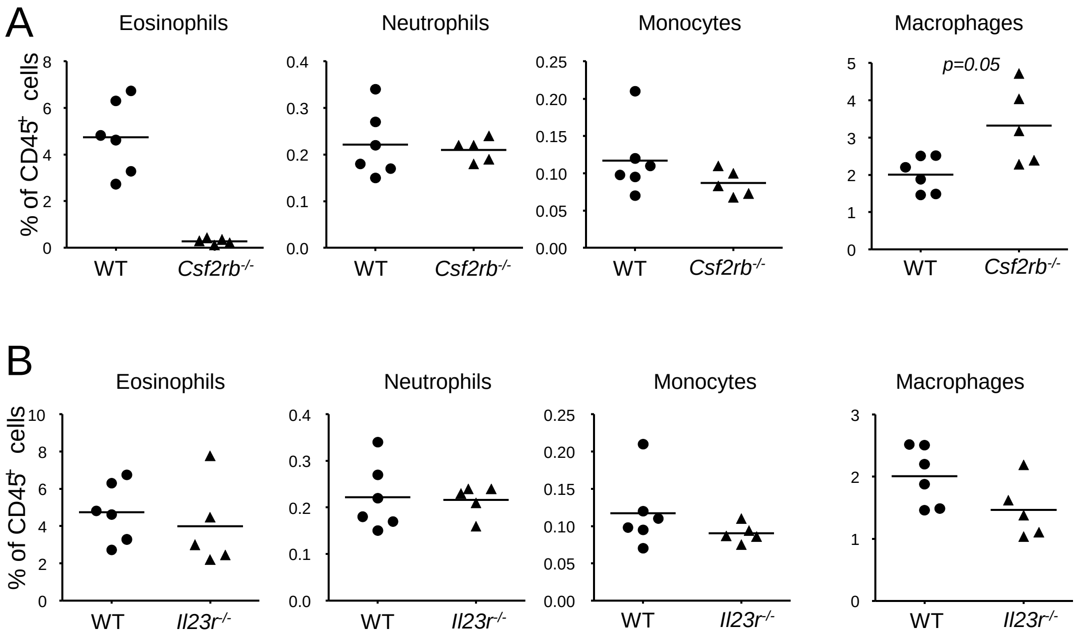

Figure S3 (related to Figure 3). Colonic myeloid cell composition in *Csf2rb*$^{-/-}$ and *Il23r*$^{-/-}$ mice at steady state

(A-B) Frequencies of eosinophils, neutrophils, MHCII^{+} monocytes and F4/80 macrophages among cLPL in C57BL/6 WT compared to *Csf2rb*$^{-/-}$ mice **(A)** and *Il23r*$^{-/-}$ mice **(B)**. Data points represent individual mice and bars represent means. Data are representative of two independent experiments.

Figure S4

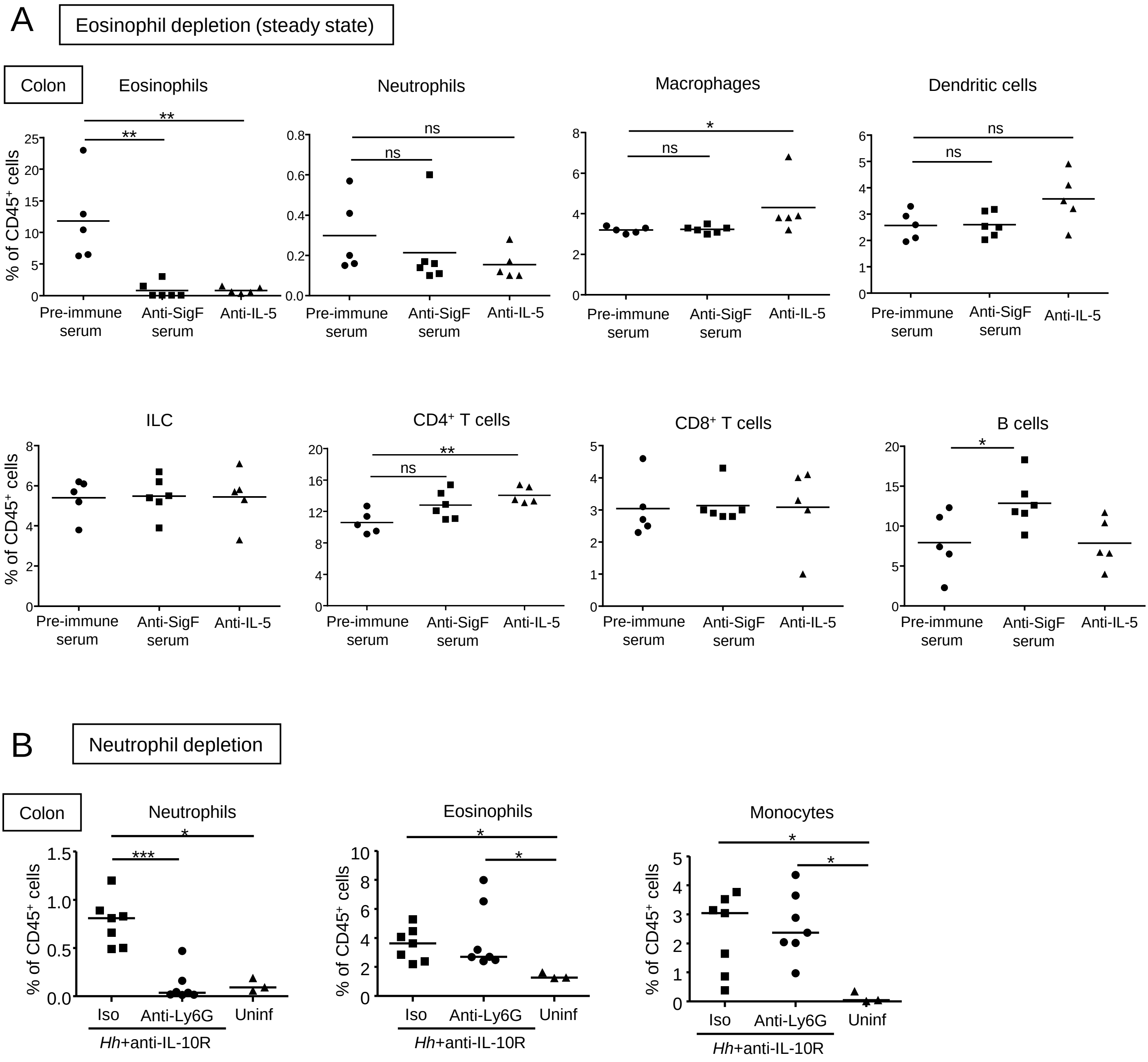

Figure S4 (related to Figure 4). Eosinophil depletion at steady state and neutrophil depletion in colitis

(A) C57BL/6 uninfected mice received two injections per week of sheep anti-Siglec-F serum or pre-immune serum or anti-IL-5 and where analyzed after 2 weeks. Frequencies of the indicated cell populations among colonic lamina propria leukocytes (cLPL) are shown. **(B)** C57BL/6 mice were infected with *H.hepaticus* (*Hh*) combined with anti-IL-10R treatment and three injections per week of anti-Ly6G or isotype control mAbs. Animals were analysed 2-3 weeks after the induction of colitis and compared to unmanipulated controls. Representative frequencies of neutrophils, eosinophils and MHCII$^+$ monocytes among cLPL are shown. Data points represent individual mice and horizontal bars represent group means. Data are representative of two independent experiments.

Figure S5

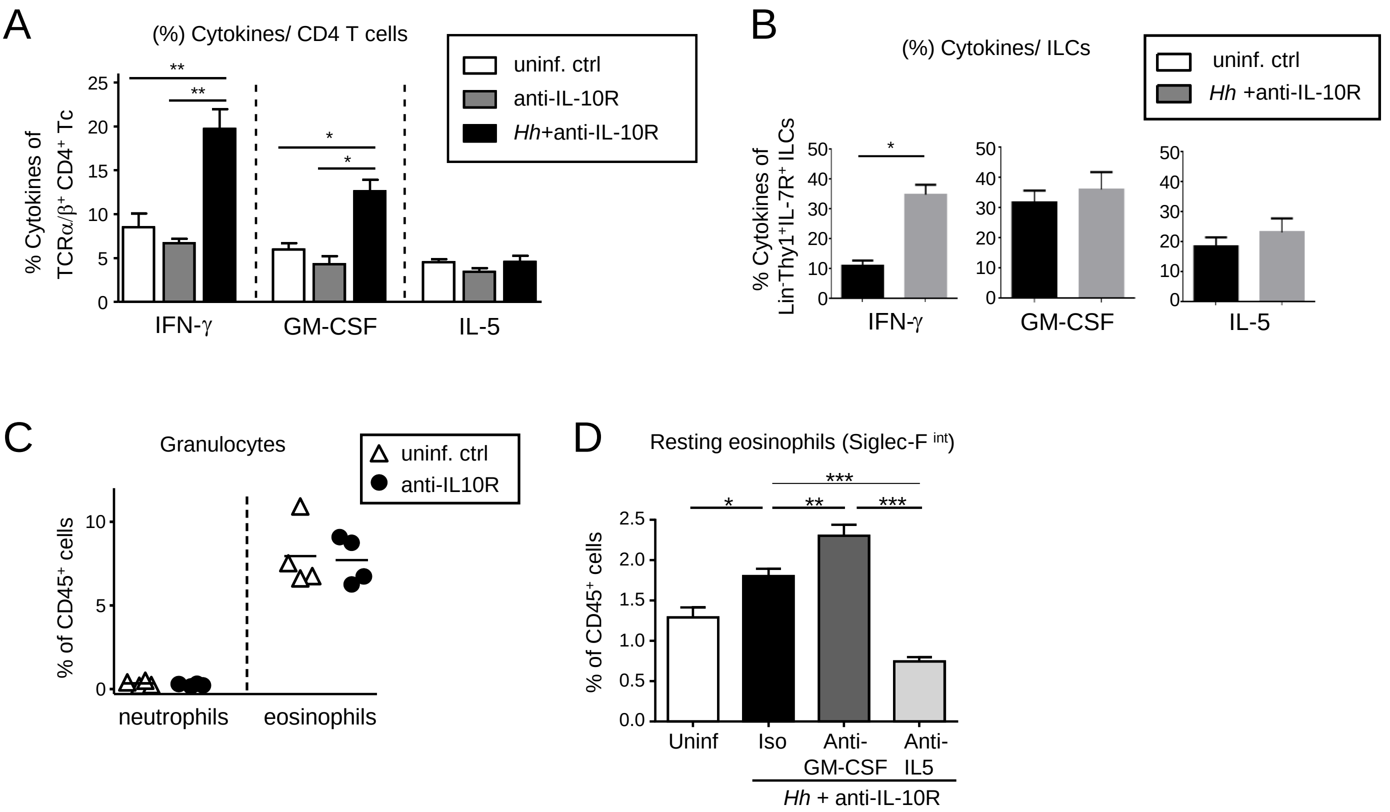

Figure S5 (related to Figure 5). Cytokine expression by CD4⁺ T cells and ILC in colitis

(A-B) C57BL/6 wt mice were infected with *H.hepaticus* (*Hh*) combined with anti-IL-10R treatment. Mice were analysed 2-3 weeks after the induction of colitis. Colonic lamina propria leukocytes (cLPL) were restimulated 4 hours with PMA/ionomycin/Brefeldin A to assess intracellular cytokines expression. **(A)** Frequencies of cytokine-expressing cells among $CD4^+$ $TCR\alpha/\beta^+$ T cells. *Hh*+anti-IL10-R treated mice were compared to uninfected controls ("uninf. ctrl") or uninfected controls only treated with anti-IL-10R antibodies ("anti-IL10R"). **(B)** Frequencies of cytokine-expressing cells among $CD45^+Lin^-$ $Thy1^+$ $IL7R^+$ innate lymphoid cells (ILCs).

(C) Frequencies of neutrophils and eosinophils among total cLPLs in uninfected mice that were either untreated (uninf. ctrl) or treated with anti-IL-10R antibodies during 2 weeks. **(D)** Mice were infected with *Hh* combined with anti-IL-10R treatment and two injections per week of anti-IL-5, anti GM-CSF or isotype control mAbs. Mice were analysed 2-3 weeks after the induction of colitis and compared to uninfected and untreated controls (uninf). Frequencies of $SiglecF^{int}$ resting eosinophils among $CD45^+$ cLPL are shown. Data points represent individual mice and horizontal bars represent group means. Data are representative of two independent experiments.

Supplemental experimental procedures

Antibodies, flow cytometry and cell sorting. For surface staining, cells were incubated at 4° C for 30 min in PBS, 0.1% BSA, 5 mM EDTA buffer containing a fixable viability dye (eBioscience) and a combination of antibodies. Staining of mature leukocytes: CCR3 (J073E5), CD11b (M1/70), CD25 (3C7), CD4 (RM4-5), CD45 (30-F11), CD45.1 (A20), CD45.2 (104), CD45RB (16A), CD63 (NVG-2), CD64 (X54-5/7.1), F4/80 (BM8), Gr1 (clone RB6-8C5), IL-33R (DJ8), IL7R (SB/199), Ly6C (HK1.4), Ly6G (1A8), MHCII (M5/114.15.2), Siglec-F (E50-2440), TCRβ (H57-597), α4β7 integrin (DATK32) and isotype controls. Staining of GMPs and EoPs: c-Kit (2B8), CD16/32 (2.4G2), CD34 (RAM34), CD45 (30-F11), IL-5Rα (T21), Sca1 (D7) and Lineage: CD3, CD4, CD8α, NKp46, B220, CD11c, Gr1, Ter119, FcεRIα.

For intracellular cytokine staining, cell suspensions were restimulated during 4 hours with PMA/ionomycin/Brefeldin A at 37° C in a humidified incubator with 5% CO_2. Cells were stained for viability and surface markers as described above, fixed and permeabilised with the (Fix/Perm and permeabilization buffer from eBioscience) according to the manufacturer's instructions. Cells were stained for 50 minutes with antibodies to GM-GSF (MP1-22E9), IL-5 (TRFK5), IFNγ (XMG1.2) and TNFa (MP6-XT22). All antibodies were obtained from eBioscience, BD Biosciences or BioLegend. Annexin V staining was performed with the AnnexinV Apoptosis Detection Set (eBioscience) according to the manufacturer's instructions. All cells were analysed with an LSRII or Fortessa (BD Biosciences) or sorted on a FACSAriaIII (BD Biosciences) to a purity of >98%. Fluorescence minus one (FMO) controls were used for gating. Analysis was performed using FlowJo software (Tree Star, Inc).

Quantitative qPCR. Homogenization of frozen colonic samples was performed using a Fastprep 24 Homogenizer (MP Biomedicals). RNA was also isolated from total colonic tissue, colonic lamina propria cells (cLPL) or from FACS-sorted cells using the RNeasy Mini kit (QIAGEN) according to manufacturer's instructions, including an on-column DNase I digestion step. cDNA synthesis was performed using the High Capacity cDNA Reverse Transcriptase (Applied Biosystems, Life Technologies). Quantitative PCR reactions for the candidate genes were performed using TaqMan gene expression assays (Life Technologies). cDNA samples were analysed in duplicate using the CFX96 detection system (Bio-Rad Laboratories) and gene expression levels for each sample were normalized to HPRT. Mean relative gene expression was determined, and the differences were calculated using the 2ΔC(t) method. Primer pairs and probes for Taqman (Life Technologies): TaqMan Gene Expression Assays for mouse *Hprt* (Mm01545399_m1), *Il5* (Mm00439646_m1), *Csf2* (Mm00432686_m1), *Ccl11* (Mm00441238_m1), *Ccl5* (Mm01302427_m1), *Tnf* (Mm00443258_m1), *Il13* Mm00434204_m1 and *Il6* (Mm01302427_m1).

Quantitation of cytokine levels, EPO and EPO activity. Mid-colon sections (one section per mouse) were weighted and cultured in RPMI 1640 medium with 10% FCS and 100 U/ml penicillin/streptomycin for 24 hours at 37° C in a humidified incubator with 5% CO_2. Cell-free supernatants were stored at -80° C and normalized to initial tissue weight in subsequent assays. Cytokines were quantified using the Flowcytomix Multiplex system (Life Technologies) according to the manufacturer's instructions and analysed by flow cytometry. Eosinophil peroxidase (EPO) was detected in supernatants by Sandwich/Capture ELISA (anti-EPX capture antibody clone MM25-429.1.1; biotylated anti-EPX detection antibody clone MM25-82.2.1) as described previously (Ochkur et al., 2012). EPO enzymatic activity was evaluated as described previously (Ochkur et al., 2012). Briefly, 50μl of supernatants were combined with 75μl of OPD-substrate containing 50mM Tris-HCl pH 8, 0.1% Triton X-100, 8.8mM H_2O_2 and 6mM KBr and 10mM o-phenylenediamine (ODP) and incubated at 37° C for 30 minutes. The reaction was terminated by the addition of 50μl of 2N H_2SO_4 to each well and read at 492 nm. Where indicated, EPO enzymatic activity was assessed on the supernatant of colonic faeces, as described previously (Forbes et al. 2004). Briefly, total colonic content was flushed with 1ml of PBS and vortexed vigorously for 5 min at 4° C. Solid faecal material was centrifuged at 10,000 × g for 10 min at 4° C and cleared supernatants were filtered and stored at −80° C until analysis.

Generation of bone marrow chimera. B6 CD45.1 mice were irradiated twice with 5.5 Gy and reconstituted i.v. with an equal number of BM cells from WT CD45.1 and *Csf2rb*$^{-/-}$ CD45.2 mice (5x10^6 cells/ mouse). Mice were rested for 8 weeks before being used in experiments.

BrdU pulse chase assay. BrdU labelling of eosinophils was performed as previously described (Ohnmacht et al., 2007), with modifications. Briefly, mice were treated with a single injection of 1 mg BrdU (5-bromo-2-deoxyuridine; BD Pharmingen) i.p. for short-term labelling (16 h) or with 3 injections of 1 mg at 12 hours interval for long-term labelling (84h). To assess BrdU incorporation, bone marrow and colonic lamina propria eosinophils were stained for CD45, CD11b, MHCII, Ly6G and Siglec-F along with a viability dye, fixed and permeabilized using the BD BrdU Flow Kit (BD Pharmingen) according to the manufacturer's instructions. Cells were incubated at 37° C for 60 min in 30 µg of DNase, followed by staining with anti-BrdU-FITC for 40 min, washed and analyzed by flow cytometry.

Eosinophil stimulation *ex vivo*, cytospins and cell size evaluation. Live CD45$^+$ CD11b$^+$ Ly6G^{lo} MHCII$^-$ SSC^{hi} eosinophils were sorted in duplicate by flow-cytometry from the colonic mouse lamina propria and seeded in round-bottom 96-well plates at a density of 50 000cells/well in IMDM medium supplemented with 10% FCS and 100 U/ml penicillin/streptomycin. Eosinophils were stimulated with recombinant mouse IL-5 (10ng/ml) or a combination of IL-5 and GM-CSF (both at 10ng/ml) and cultured for 16-18 hours at 37° C in a humidified incubator with 5% CO_2. Cells were then lysed for mRNA extraction as described above or cytocentrifuged onto glas slides (cytospins) and stained with the Hemacolor® Rapid staining of blood smear staining set (Merck). Eosinophils were visualized with ImageJ open access software to determine the average diameter value per cell.

Immunofluorescence. For immunofluorescence, samples of the mid colon were taken and immediately embedded in OCT compound (Tissue-Tek) and frozen in a bath of isopentane on dry ice prior to storage at -80ºC. 6µm frozen sections were cut and collected on frosted glass slides. For staining, slides were fixed in 2% formalin. Endogenous peroxidase activity was blocked with 1% H_2O_2 (Sigma) and 2% sodium azide and non-specific binding was blocked with 10% donkey serum. Where indicated sections were incubated with anti-SiglecF (E50-2440, BD Biosciences) in 10% donkey serum. Sections were then incubated with donkey anti-rat FITC secondary antibody (Jackson ImmunoResearch Laboratories). Alternatively sections were incubated with Ly6G-APC (1A8, eBioscience). Sections were mounted with Vectashield containing DAPI. Images were collected using a 710 microscope (Carl Zeiss), and observed using ImageJ open access software.

Supplemental reference

Ochkur S.I., Kim J.D., Protheroe C.A., Colbert D., Moqbel R., Lacy P., Lee J.J., Lee N.A. (2012). The development of a sensitive and specific ELISA for mouse eosinophil peroxidase: assessment of eosinophil degranulation ex vivo and in models of human disease. J Immunol Methods, 375, 138–147.
